# Evolutionary Traits that Enable Scleractinian Corals to Survive Mass Extinction Events

**DOI:** 10.1038/s41598-020-60605-2

**Published:** 2020-03-03

**Authors:** Gal Dishon, Michal Grossowicz, Michael Krom, Gilad Guy, David F. Gruber, Dan Tchernov

**Affiliations:** 1 Department of Marine Biology, The Leon H. Charney School of Marine Sciences, University of Haifa, Mount Carmel, Haifa 31905 Israel; 2 Scripps Institution of Oceanography, University of California, San Diego, La Jolla, CA 92093 USA; 3 GEOMAR Helmholtz Centre for Ocean Research, Kiel, Germany; 4 Morris Kahn Marine Research Station, Environmental Geochemistry Lab., Leon H. Charney School of Marine Sciences, Haifa University, Mount Carmel, Israel; 5 School of Earth and Environment, University of Leeds, Leeds, LS2 9JT United Kingdom; 6 Department of Natural Sciences, Baruch College, City University of New York, New York, NY 10010 USA; 7 PhD Program in Biology, The Graduate Center City University of New York, New York, NY 10010 USA

**Keywords:** Ocean sciences, Ecology, Marine biology

## Abstract

Scleractinian “stony” corals are major habitat engineers, whose skeletons form the framework for the highly diverse, yet increasingly threatened, coral reef ecosystem. Fossil coral skeletons also present a rich record that enables paleontological analysis of coral origins, tracing them back to the Triassic (~241 Myr). While numerous invertebrate lineages were eradicated at the last major mass extinction boundary, the Cretaceous-Tertiary/K-T (66 Myr), a number of Scleractinian corals survived. We review this history and assess traits correlated with K-T mass extinction survival. Disaster-related “survival” traits that emerged from our analysis are: (1) deep water residing (>100 m); (2) cosmopolitan distributions, (3) non-symbiotic, (4) solitary or small colonies and (5) bleaching-resistant. We then compared these traits to the traits of modern Scleractinian corals, using to IUCN Red List data, and report that corals with these same survival traits have relatively stable populations, while those lacking them are presently decreasing in abundance and diversity. This shows corals exhibiting a similar dynamic survival response as seen at the last major extinction, the K-T. While these results could be seen as promising, that some corals may survive the Anthropocene extinction, they also highlight how our relatively-fragile Primate order does not possess analogous “survival” characteristics, nor have a record of mass extinction survival as some corals are capable.

## Introduction

Scleractinian corals represent an ideal taxon to serve as a model for describing past and predicting future environmental trajectories of mass extinctions. Coral skeletons are widespread and well-preserved in the fossil record, and as reef-builders, Scleractinians support and promote biodiversity hotspots. While coral reefs represent only 0.2% of the oceans’ area, they harbor ~95,000 described species and represent about 5% of the world’s known species and ~35% of known marine species^1^. For these reasons they have been extensively studied and relatively well-monitored over the past few decades^2,3^. The International Union for Conservation of Nature (IUCN) Red List has recently reported an alarming trend, ca. 30% of the 96,500 species assessed on the IUCN Red List being threatened with extinction^4^, including one third of the world’s corals^5^.

The earth has experienced five major mass extinction events (“The Big Five”) since the Cambrian period, each resulting in the loss of more than three-quarters of species biodiversity over a geologically short time interval. These events occurred at the 1) Late Ordovician (440 Mya), 2) Late Devonian (370–350 Mya), 3) End-Permian (251 Mya), 4) End-Triassic (201 Mya) and 5) End-Cretaceous, which is referred to as the ‘K-T’ event (66 Mya). It has been proposed that the sixth mass extinction is currently in progress^6^ with increasing number of studies confirming that current extinction rates are more than 100 times higher than background extinction rates^7^. This rate of species loss is on par with previous extinction events^6,8–10^. This study tests the hypothesis of common evolutionary traits/dynamics that characterize both K-T and the current Anthropocene survivors. We use Scleractinian corals as the test subject due to their secretion of a calcium carbonate skeleton that provides a rich history for paleontological analysis that extends back to the Triassic period^11,12^.

### The End-Cretaceous (K-T) mass extinction

The K–T mass extinction was one of the most destructive events in the Phanerozoic, resulting in global extinction of ~40% of total genera and 47% of marine invertebrate genera^13^. It is widely accepted that this extinction was triggered by a giant (10 km in diameter) meteorite impact at Chicxulub, Yucatan Peninsula, Mexico 66 million years ago^14^. The bolide event likely caused earthquakes, tsunamis and intense heat pulse that led to global wildfires, which had a strong impact on non-marine organisms^15^. The initial “fireball stage” was followed by a global “impact winter” caused by dust particles and other aerosols blocking sunlight, resulting in a 6 °C cooling of sea surface temperature. The impact winter lasted months to decades and influenced both marine and terrestrial productivity, leading to nutrient soup accumulations, and the formation of stable cold deep water^16^. In addition, sulfur and nitrogen volatile compounds were injected to the atmosphere, possibly leading to acid rain and ocean acidification^17^. The acid rain increased chemical weathering rates and resulted in a higher flux of bioavailable phosphorus into the ocean, resulting in additional nutrients to the nutrient soup^18^. The K-T boundary catastrophe reached deep-water dwellers, inferred by significant deterioration of deep-water benthic foraminifera communities^19^. After the relatively short-lived cool impact winter, rapid warming brought sea surface temperature (SST) back to pre-K-T levels followed by long-term warming, apparently related to greenhouse gases release^20^. Some other scenarios for the K-T event drivers include volcanism, multiple asteroid impacts, climatic changes and biotic stresses already affecting organisms previous to the K-T, as well as combinations of these factors^9^. Recent modeling experiments predict that the “impact winter” might have been balanced, or even outweighed, by global warming derived by water vapors, leading to a greenhouse effect^21^. Scleractinian corals are expected to be particularly sensitive to bespoke environmental changes such as: (1) prolonged darkness, (2) major temperature changes, (3) eutrophication and (4) ocean acidification.

The recovery of coral reefs following the K-T event began with coralline algae, sometimes accompanied by photosymbiotic benthic foraminifera. While reef-building photosymbiotic corals suffered great losses at the End-Cretaceous, some genera survived and later became important reef builders in the Cenozoic^22^. Coral reefs were re-established ~2–5 Ma after the K-T, and became increasingly abundant in the Eocene (~10 Ma later). These reefs were composed of mostly novel community types, compared to previous Cretaceous reefs^22^.

### The Anthropocene extinction

*Homo sapiens* first appear in the fossil record ~315,000 years ago^23^ and since that time there have been several regional extinctions, becoming more frequent following the agricultural/neolithic revolution beginning ~10,000 years ago^9^. However, since the Industrial Revolution (1760–1840) and particularly after 1950, the Earth’s biosphere has experienced rapid changes in atmospheric conditions, warming of global temperatures and rising CO_2_ levels. These are the highest levels over the past 800,000 years and temperatures are predicted to increase by ~1.8–3.4 °C and CO_2_ levels by 170–420 ppm over the next century^24,25^. Increased atmospheric CO_2_ concentrations are causing measurable ocean acidification^26^. This together with habitat fragmentation, pollution, overfishing and overhunting, the introduction of invasive species and pathogens, and expanding human biomass represents a combination of global wide extreme stressors combining in unprecedented ways for most species^6,27^. If these stressors are not mitigated, 75% of species are predicted to face extinction within the next few centuries to millennia^6^. Stony corals are particularly sensitive and are heavily impacted by direct human interferences (e.g. overfishing, pollution, coastal development and tourism damage), as well as warming and ocean acidification. They are predicted to suffer more than 30–50% extinction in the coming decades and century, respectively^5,24^.

### Current extinction risk assessment

Realizing that extinction risk is realistic to increasing number of species in our era, the International Union for Conservation of Nature (IUCN) initiated the Red List of Threatened Species in 1964. It aimed to classify organisms at extinction risk in categories that may assist in managing conservation efforts efficiently. The Red List is recognized as the most comprehensive assessment of organisms’ extinction risk^28^ with species assigned categories, from “extinct” to “of least concern”. This assignment is performed by taxonomic experts according to the IUCN Red List categories and criteria^29^ that consider reports of population reduction, as well as threats and risk factors such as bleaching/disease susceptibility, severe fragmentation and destruction of habitat. In this study, we used data from the coral fossil record and the current IUCN Red List classification to compare coral survival between the K-T and the current extinction events.

## Methods

Fossil occurrence data for Anthozoans (genus, first and last time of occurrence, geographic position, coloniality and photosymbiosis) were collected from the Paleobiology Database (http://paleobiodb.org). These data were joined with various extant coral traits including the IUCN Red List classification from the Coral Trait Database (https://coraltraits.org). Data were grouped at the genus level in order to allow robust comparison with the fossil record and overcoming some uncertainties related to species level recognition.

Trends in coral traits were extracted from the fossil record by querying the combined database of coral fossil occurrence and genus traits. From this database, the last 250 million years were analyzed at 5 Myr intervals (e.g. from all the coral genera found between 40 to 45 Mya, the percentage of colonial genera was calculated to give “coloniality prevalence”). Results were further analyzed if the number of genera in a bin was >10. The analysis included binning of data into 5 M years bins and then calculating the percentage or average (for binary and numerical traits accordingly) of each trait in each time bin.

For this analysis, each fossil that has an extant representative at the genus level (even if the species is extinct) was assumed to be characterized with the same traits as its extant representative. Using this approach, we could infer relevant traits that were not evident in the fossil record (e.g. bleaching and disease susceptibility). Since only ~10% of extant Scleractinian genera are found earlier than the K-T boundary (22 of 223 genera in the database), the traits that were inferred from modern corals possessed smaller representation than those from fossils (e.g. at 66 Mya, 12 genera were classified as bleaching tolerant/susceptible, while 74 genera were classified as colonial/solitary based on previous fossil classification^30^). Although adaptation of a coral’s population to environmental conditions is a known phenomenon and plasticity of bleaching susceptibility has been documented for some species^31^, a significant change in a genus bleaching susceptibility is uncommon^32^. “Winners and losers” taxa are indicated for bleaching episodes^33–35^ often related to consistent morphological features^36,37^. Hence, we assumed there would be differences in bleaching and disease susceptibility among genera even if some adaptation takes place.

### Multivariate analysis

The characteristic binary traits of 102 coral genera, for which the full suite of metadata was available (Table 1), were analyzed here with 2D non-metric multidimensional scaling (nMDS) ordination. The ordination was grouped by K-T boundary survival (“survivors” and “extinct”), and Red List category (which represents whether a genus survived the K-T event and its estimated risk for modern extinction). The data were first standardized using Gower transformation prior to nMDS analysis, and Manhattan distance was used for the dissimilarity distances matrix. Stress value, which represents the divergence of the real value from the ordination output^38^, was calculated. Stress values lower than 0.2 mean that the ordination is useful, but higher values (>0.1) should be analyzed with caution. One-way Analysis of Similarities (ANOSIM) was performed to statistically differentiate the characteristic binary traits of the grouping (“survivors” and “extinct”). This test is a-parametric and does not assume normality of the data. The calculated test statistic R has a value between −1 and 1 and rarely goes below 0^38^. When R = 1, all the repeats within a group are similar to each other, rather than to repeats in other groups. When R = 0, the similarity within and among all groups is the same on average. All values are presented at a confidence interval of 95%. Similarity Percentage Analysis (SIMPER) identifies the “important” component from all the characteristic binary traits; i.e., the relative contribution of each trait to the dissimilarity between all inter-group pairs of samples. All multivariate analyses were performed with R i386 3.3.3.

**Table 1:** List of traits used for nMDS analysis.

Trait	Value	Description
Global distribution rank	1–5	Number of ocean basins a genus was found in
Upper depth	0–373 m	Most shallow depth recorded for genus
Lower depth	5–2165 m	deepest depth recorded for genus
Zooxanthellate	0/1	Genus host photosymbionts
Water clarity preference	Clear/Turbid	Genus occurred at clear/turbid waters
Wave exposure	Protected/Exposed	Genus occurred at protected/exposed habitats
Colonial	0/1	Genus forms colonies
K-T survival	0/1	Genus were found before and after the K-T event
Red List category	1–5	Contemporary extinction risk (1 = Least Concern, 2 = Near Threatened, 3 = Vulnerable, 4 = Endangered, 5 = Critically Endangered.

## Results

### Comparing the K-T and Anthropocene extinction events

**Figure 1:**
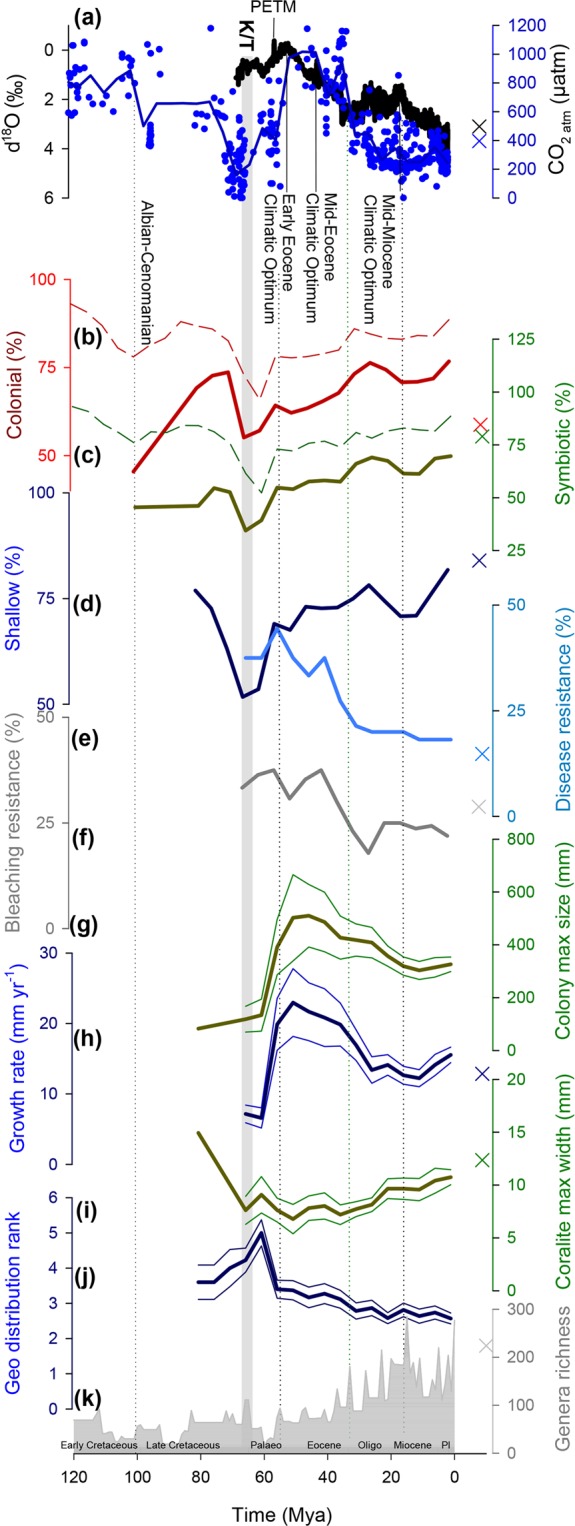
The prevalence of coral’s traits throughout the past 120 Mya based on fossil coral occurrences and corresponding traits. (**a**) Paleo-climate context is given with SST (as δ^18^O)^62^ and smoothed atmospheric CO_2_^63^. (**b–j**) Dynamics of coral traits throughout the past 120 Mya. Data are integrated over 5 Myr intervals and include only results where the number of genera exceeds 10. Colored “X” symbols on the right represent current status of the examined traits. (**k**) Gray area along the x-axis shows Scleractinian genera diversity in the fossil record. Solid lines represent traits inferred from extant coral genera, whereas dashed lines represent traits derived from the coral fossil record (based on the KTbase^30^). Thin lines represent confidence intervals for traits that have numerical value (**g–j**).

Examination of various coral traits represented in the fossil record showed pronounced features related to the K-T mass extinction event (Fig. 1). These trends include a 18% decrease in coloniality, a 18% decrease in photosymbiosis (in agreement with previous studies^30,39,40^) and a 12% decrease in the occupation of shallow habitats (depth <100 m) relative to pre K-T values. We also note a higher prevalence of slow growing genera (average growth rate <10 mm yr^−1^) around the K-T event (89% at the late Cretaceous as opposed to 60% at the Pliocene-Pleistocene), manifested also as slow average growth rate (~7 mm yr^−1^) at 60–66 Mya (Fig. 1). All the declines in these traits are followed by distinguishable increases following the K-T extinction event (10% in coloniality, 20% in photosymbiosis and 17% in shallow habitats occupation). In addition, the average of maximal colony size increased just after the K-T event (from 118 mm at 66 Mya, before the K-T, to 391 mm at 55 Mya). By contrast, bleaching and disease-resistance were high (36% and 37% respectively) during the K-T event, but remain in stable proportions until ~40 Mya, where a drop was recorded for both (overall decrease of 15% and 19% respectively), coinciding with a general Sea Surface Temperature (SST) cooling trend. Interestingly, representations of most traits in the modern coral inventory (Fig. 1, “X” symbols) show a recent change (compared with the most recent fossils), with trends that are similar to those found associated with the changes through the K-T event (e.g. decreasing coloniality, photosymbiosis and colony size).

**Figure 2:**
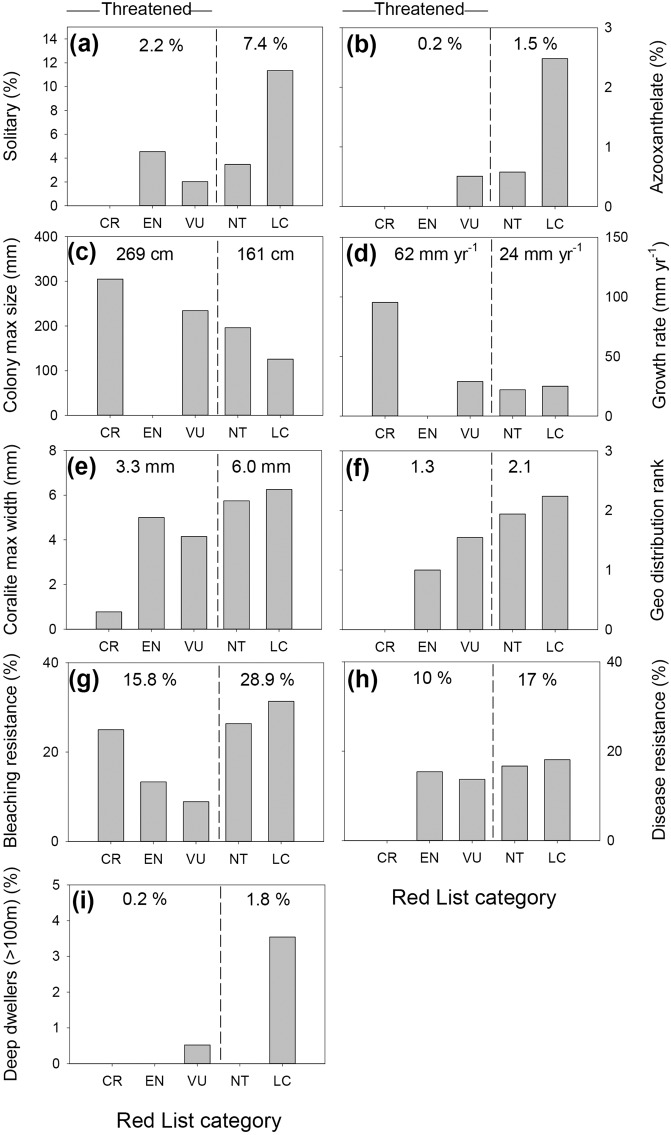
Current representation of coral traits in the IUCN Red List categories. Analyses were performed at the species level based on data from CoralTraits database. Red list categories VU, EN and CR are classified as “threatened” and averages are written for “threatened” and LC + NT categories. Numbers at top are averages of threatened/unthreatened categories.

In order to assess the trends in coral traits characterizing the modern extinction, we used the IUCN Red List, which determines the relative risk of extinction for a wide taxonomic range. This consists of seven groupings: 1) “Extinct”; 2) “Extinct in the Wild”; 3) “Critically Endangered”; 4) “Endangered”; 5) “Vulnerable”, 6) “Near Threatened” and 7) “Least Concern”. Our analyses showed that some traits were less represented in categories 1–5 (Extinct-Vulnerable) than in 6–7 (Near Threatened-Least Concern) categories (Fig. 2).

We find that corals less endangered by the current extinction are solitary (7.4% of all species in “Least Concern” and “Near Threatened” categories vs. 2.2% in the Threatened Red List categories); do not contain zooxanthellae (1.5% in “Least Concern” and “Near Threatened” vs. 0.2% in the Threatened Red List categories); and build only small colonies (<200 mm) (averaged 269 cm in Threatened Red List categories vs. 161 cm in “Least Concern” and “Near Threatened”). The less endangered corals have larger corallites (>3 mm) (averaging 3.3 cm in Threatened Red List categories vs. 6.0 cm in “Least Concern” and “Near Threatened”), are slow-growers (<50 mm yr^−1^) (averaging 62 mm yr^−1^ in Threatened Red List categories vs. 24 mm yr^−1^ in “Least Concern” and “Near Threatened”); and are capable of occupying deep reefs (>100 m depth) (1.8% in “Least Concern” and “Near Threatened” vs. 0.2% in the Threatened Red List categories). Of the corals reported to be sensitive or resistant to disease, all of the “Critically Endangered” were disease-susceptible. Bleaching resistance was found to be in the highest proportion (31%) within the “Least Concern” category, but among the “Threatened” categories it was higher in the “Critically endangered” than in “Vulnerable” categories (25% and 9% respectively).

**Figure 3:**
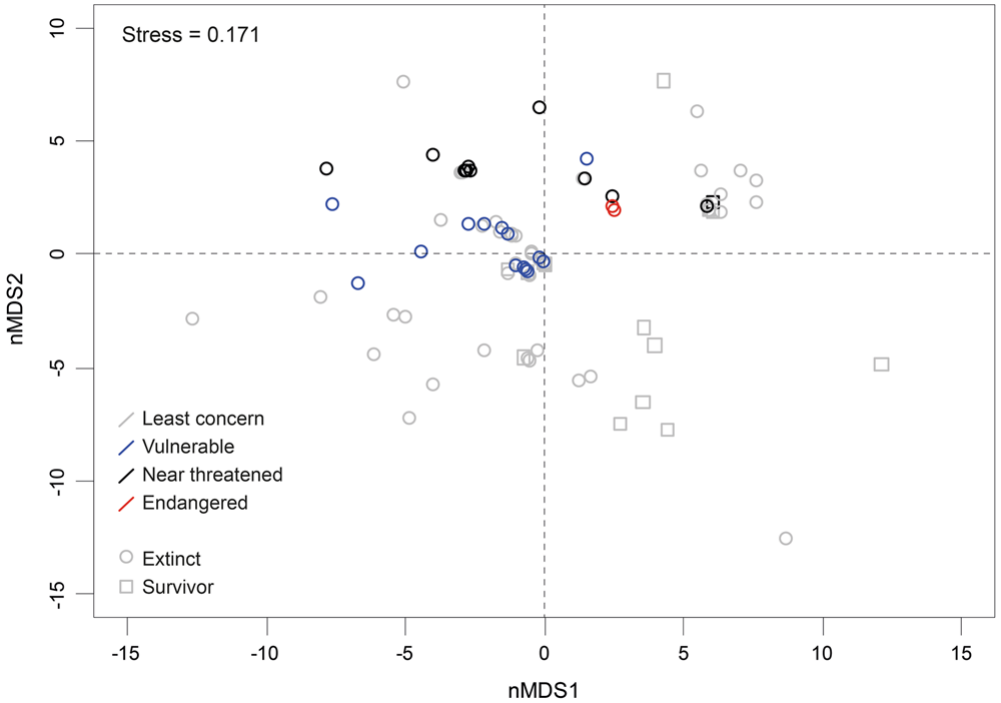
nMDS analyses of coral genera. Each circle represents a coral genus and its coordinates are representing this coral’s traits. The color refers to IUCN Red List category and the shape (square/circle) refers to its survival/extinction through the K-T extinction.

**Figure 4:**
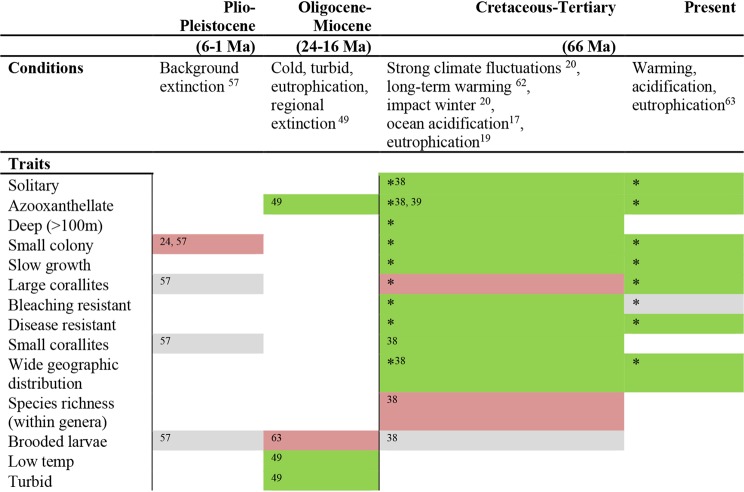
Summary of the traits characterizing extinction survivors. Colors of cells represent whether trait is favored (green), rejected (red), or no influence (grey). Asterisks (*) represent findings from this study.

The global distribution rank (calculated as the sum of ocean basins where a genus was found) was highest through the K-T event (Fig. 1), implying an advantage for cosmopolitan genera during this event. This rank is also higher with genera having reduced extinction risk today (Fig. 2). nMDS analysis (stress = 0.171) showed that the surviving corals’ traits were different than the non-surviving ones. This difference is shown to be statistically significant (ANOSIM, global R = 0.25, *p *< 0.01) (Fig. 3). SIMPER analysis shows that geographic distribution characteristics contributed the most to whether a group survived or became extinct at the K-T event. Combined with the “Global rank” trait, the geographic traits explain 59% of the dissimilarity (Table 2). Furthermore, wide geographic distribution was noted as high determinant of genus survival both for K-T survivors and present “Least Concern” genera. A summary of the coral traits that survived through the K-T mass extinction and are at lower risk for modern extinction can be found in Fig. 4.

**Table 2:** SIMPER results for trait contribution to dissimilarity between “survivors” and “extinct”. Cumulative sum represents the cumulative contribution of a specific trait together with the preceding traits.

Traits	Contribution	Cumulative sum
Eastern Atlantic	0.131	0.131
Global rank	0.111	0.241
Western Atlantic	0.103	0.344
Indian Ocean	0.091	0.435
Eastern Pacific	0.089	0.523
Western and Central Pacific	0.069	0.593
Min. of upper Depth	0.066	0.658
Max. of lower Depth	0.065	0.723
No Zooxanthellae	0.059	0.782
Water clarity preference: clear	0.050	0.832
Water clarity preference: turbid	0.045	0.877
Wave exposure: exposed	0.044	0.921
Solitary	0.037	0.958
Colonial	0.030	0.988
Wave exposure: protected	0.012	1

## Discussion

Deducing future trajectories, based upon past extinction events, has received increased scientific attention over the past decade^41–44^. Understanding the effects of biological traits on coral survival and future community structure is a matter of high priority^41,45^, both for what it predicts for the future of coral reefs and as a general model for the selective survival of various biological communities. In this study we searched the existing body of coral fossil data and coral traits through the K-T boundary event, together with their contemporary extinction status, to determine whether there are “survival traits” that are common both among survivors of the last major mass extinction (K-T). and at the present Anthropocene mass extinction.

We found wide geographical distribution to be the most important determinant of coral survival both through the K-T and the Anthropocene (Table 2, Fig. 2). This finding may reflect a geographic heterogeneity of these extinction events elevating survival chances for widespread genera. Wide geographical distribution was also noted as the only trait significantly characterizing bivalves survival during mass extinctions^46,47^. However, this result should be taken with caution regarding the Anthropocene extinction as it is based on analyzes of the IUCN Red List categories whose assessment might also take geographical distributions parameters into account.

Our analysis shows that colonial, symbiotic, shallow Scleractinian genera were particularly impacted by the K-T extinction, a finding that is in agreement with^40^. We also show that these genera evolved in higher proportions following the extinction (Fig. 1) and are now the main contemporary tropical coral reef builders. However, this origination through the Tertiary is currently in jeopardy, with many colonial, symbiotic genera falling within the modern IUCN Red List threatened-extinction categories (Fig. 2) and community shifts are taking place over large reef areas that favor slow growing corals^34^. This transition may have previously occurred^48^, as it’s suggested that photosymbiotic colonial corals were disproportionately removed during the Triassic extinction^45^, the mid Cretaceous^40^ and the Oligocene-Miocene extinction^49^. The sensitivity of the closely-related traits (colonial, symbiotic and shallow-dwelling)^39^ to K-T and Anthropocene extinctions may be explained by the combined effect of eutrophication, warming and acidification which characterize both the modern situation and some parts of the K-T event^50^. These environmental changes in the modern ocean impact mostly euphotic surface waters and are particularly threatening to colonial symbiotic genera that reside in the upper, photic ocean, where they outcompete their algae competitors in low-nutrients environments. An alternative hypothesis is that Scleractinian corals retreated into deeper, off-reef niches where light was scarce^40^. In the deep reef environment, non-photosymbiotic solitary coral genera have an advantage, while symbiotic corals suffered higher extinction rates on shallow reef habitats during the K-T^40^. Decreasing average growth rates through the K-T event may also be related to the drop of photosymbiosis which would diminish growth rates due to lesser energy and photosynthates supply^51^. Decreasing growth rates may also reflect the coinciding high bleaching resistance found during the K-T (Fig. 1) since many fast-growing corals are thin-tissue branched colonies that are suggested to be more susceptible to bleaching^33^. Our analysis reports an evolutionary selection towards slower growing genera, rather than merely a decrease in the growth rates of a genus, as documented in recent ecological studies e.g^52,53^. Therefore, while fast-growing genera have higher extinction risk, it might also be due to other accompanying traits (coloniality, symbiosis, shallow habitat occupation and branching forms).

In addition, coral genera with relatively greater disease or thermal stress resistance, were relatively dominant (~40%, ~30% respectively) in those that survived the K-T event and Paleocene and Eocene high temperatures (Fig. 1). Decreasing temperatures were later accompanied by the evolution of genera with higher proportion of vulnerable traits leading to a lower proportion (~20%) of resistant genera. This relative decrease of resistant genera during low SST periods may suggest that high SST may be a cause of increased mortality by disease and bleaching on a geologic time scale, such as following the K-T event. This finding is in accordance with^54^ that shows higher disease effects coinciding with past thermal stress history. The high proportion of bleaching resistant genera after the K-T event reinforces the suggestion that bleaching resistance is a significant survival factor during mass extinctions^39,45,55^. This finding offers another perspective to the “adaptive bleaching hypothesis”^56^, which states that when environmental conditions change, one or more clades of photosymbionts is replaced by a new symbiotic consortium of photosymbionts that are better suited to the current conditions. Interestingly, for the Anthropocene event, bleaching susceptibility was found to be in the highest proportion (91%) within “Vulnerable”, rather than in the “Critical” (75%) Red List category. This could be interpreted as the bleaching threat being not yet fully established, or that other factors are stronger in determining probable extinction patterns. A recent model for coral endurance through global warming predicts that bleaching resistance will become a dominant factor for future coral survival^41^.

There is potentially a difference in corals surviving moderate extinction (by possessing large colony size) and mass extinctions (by possessing small colony size). Ecological characteristics were previously found to be an important determinant of extinction risk in Caribbean reef-corals in the Neogene with colony size as the most important trait determining extinction rate. It was found that for the Neogene moderate extinctions, corals with small, massive colonies were most vulnerable^57^ and large colonial corals survived. A similar trend, of higher extinction risk for smaller organisms during “background extinctions” as opposed to higher extinction risk for large bodied creatures in the Anthropocene, was found for marine vertebrates and mollusks^58^. These discrepancies imply different mechanisms allowing a coral to survive during moderate versus mass extinction. In moderate extinction conditions, large colony size (associated with a higher number of polyps per colony, fragmentation capabilities and high rates of recolonization after local extinctions) allows a coral to survive some disturbances. While in mass extinction conditions, such as the K-T (this study), small colony size allows corals in “sheltered” niches to survive. This tendency of smaller coral genera to survive through a mass extinction resembles the well-known “Lilliput Effect”, a trend towards smaller size of faunal elements associated with mass extinction events^59^. In this sense, the fate of large corals is similar to that of large terrestrial vertebrates, who were drastically impacted by the K-T mass extinction event^60^.

**Figure 5:**
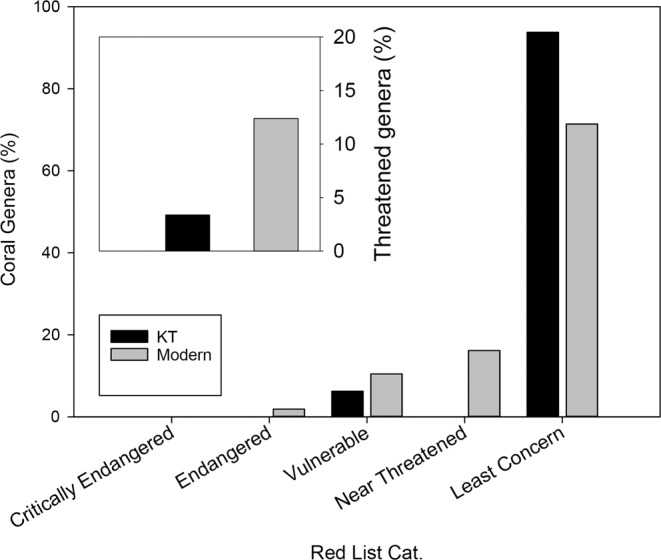
Current “extinction status” of Pre and Post K-T coral genera (grey and black bars respectively). Pre K-T corals (N = 16 genera) are those found in the fossil record before 66 Mya, Post K-T corals (N = 59 genera) only emerged later than 66 Mya. Inset, the percentage of pre and post K-T threatened genera (Red List category higher than Near Threatened). Red List category for a genus was defined as the category of the least threatened species within the genus.

While the conditions of the K-T mass extinction event were likely different from conditions of the current Anthropocene extinction, this study notes distinctive similarities between coral traits that survived the K-T mass extinction event and those that are least threatened in the current extinction event. This leads to the hypothesis that coral genera that evolved at or before the K-T event (“K-T corals” Fig. 5) may be better adapted to survive the modern extinction than genera that evolved afterwards (i.e. “Modern corals” Fig. 5). This holds true when analyzing the presence of “modern” and “K-T” genera along the IUCN Red List (https://www.iucnredlist.org) categories (Fig. 5). This analysis shows that “K-T corals” are less threatened by modern extinction, than corals that evolved after the Cretaceous period.

We suggest that the similarity in traits allowing coral survival exists through two very different extinction events (Table 2) imply that there is a basic mechanism which enables a coral to become a “mass extinction survivor” and which may be maintained for tens of millions of years. Recognizing this, we might predict a long-term change in coral assemblages to more solitary, non-symbiotic communities, similar to early Paleocene assemblages. In this scenario, already evidenced in some of the world’s largest coral reef systems^34^, the dominance of corals in contemporary coral reefs may be replaced by algae or other invertebrates’ dominance, due to the loss of competitive advantage in low nutrient exploitation and structure building. A community shift in this direction will damage their reef-building capacity and structural complexity that supports the highest biodiversity among all marine ecosystems. This study provides alarming evidence that reef communities are currently in the process of transitioning into disaster communities, akin to previous extinction events. Recovery of coral reef ecosystems from the K-T mass extinction was slow (2–10 My), as judged by the Paleocene re-establishment of algal symbiosis^39^. While the slow recovery time of coral reefs following a mass extinction is distressing, we also call attention that Primates (the Order that also includes humans) are also increasingly becoming threatened with extinction^61^. And unlike the Order Scleractinia, Primates do not possess analogous “survival” traits that enable some species to transcend major extinction boundaries, nor does *Homo sapiens* or any other Primate species, have a track record of mass extinction survival.
